# REDUCING LAPSES IN HEMOGLOBIN A1C TESTING

**DOI:** 10.51894/001c.122866

**Published:** 2024-08-30

**Authors:** Brandon Elliot, Lisa Wade

**Affiliations:** 1 Family Medicine McLaren Bay Region

## 24

### INTRODUCTION

Hemoglobin A1C is a blood test that measures average blood sugar over the past 3 months. The goal for most people with diabetes is 7% or less. However, goal A1c depends on many factors including age or other medical conditions. Closure of this healthcare gap is monitored by Healthcare Effectiveness Data and Information Set (HEDIS) and overseen by the National Committee for Quality Assurance (NCQA).

### OBJECTIVES

Identify patients at the Family Health and Wellness Center (FHWC) aged 18-75 with diabetes (Type 1 or 2), A1c measurement > 9% and last reported A1c of more than 3 months old.

### METHODS

Curated a quality report identifying FHWC members with age 18-75 with diabetes (Type 1 or 2), A1c measurement > 9% and last reported A1c of more than 3 months old. Members were contacted via telephone and/or by postcard to schedule an in-person appointment at the FHWC for an A1c point of care (POC) and optimization of diabetes management over a 3-month study period from 11/6/23 - 2/6/24.

### RESULTS

Twenty-three patients met the assigned criteria. Seven patients were no longer established at the FHWC. One patient was residing in a nursing home. One patient had A1c monitoring through a 3rd party partnership with their insurance company. Two patients no longer had insurance. Twelve patients were contacted via telephone and/or postcard to schedule appointment for POC A1c, but six did not respond to outreach. The remaining six patients were seen at the FHWC and POC A1c was obtained. Of these six patients, three had A1c measurements < 9% and three had A1c measurements that remained above 9%.

### CONCLUSION

This quality improvement (QI) was to help establish a process for HEDIS A1c monitoring of quality measures for the FHWC. McLaren Bay Region transitioned from paper charting to integrating an EHR in July of 2021. Prior to this integration, paper charts were historically marked by Quality Improvement Specialists flagging A1c measures that needed to be followed up and gaps closed. Now, an electronic process can be leveraged going forward.

